# Association of Circulating Very Long-Chain Saturated Fatty Acids With Cardiovascular Mortality in NHANES 2003-2004, 2011-2012

**DOI:** 10.1210/clinem/dgad561

**Published:** 2023-09-21

**Authors:** Xinmiao Tao, Lin Liu, Pingnan Ma, Jinxia Hu, Zhu Ming, Keke Dang, Yuntao Zhang, Ying Li

**Affiliations:** Department of Nutrition and Food Hygiene, School of Public Health, Key Laboratory of Precision Nutrition and Health, Ministry of Education, Harbin Medical University, Harbin, Heilongjiang, 150000, China; Department of Nutrition and Food Hygiene, School of Public Health, Key Laboratory of Precision Nutrition and Health, Ministry of Education, Harbin Medical University, Harbin, Heilongjiang, 150000, China; Department of Nutrition and Food Hygiene, School of Public Health, Key Laboratory of Precision Nutrition and Health, Ministry of Education, Harbin Medical University, Harbin, Heilongjiang, 150000, China; Department of Nutrition and Food Hygiene, School of Public Health, Key Laboratory of Precision Nutrition and Health, Ministry of Education, Harbin Medical University, Harbin, Heilongjiang, 150000, China; Department of Nutrition and Food Hygiene, School of Public Health, Key Laboratory of Precision Nutrition and Health, Ministry of Education, Harbin Medical University, Harbin, Heilongjiang, 150000, China; Department of Nutrition and Food Hygiene, School of Public Health, Key Laboratory of Precision Nutrition and Health, Ministry of Education, Harbin Medical University, Harbin, Heilongjiang, 150000, China; MED-X Institute, Center for Immunological and Metabolic Diseases (CIMD), First Affiliated Hospital of Xi’an Jiaotong University, Xi’an, 710000, China; Department of Nutrition and Food Hygiene, School of Public Health, Key Laboratory of Precision Nutrition and Health, Ministry of Education, Harbin Medical University, Harbin, Heilongjiang, 150000, China

**Keywords:** very long-chain saturated fatty acids, cardiovascular mortality, coronary heart mortality, all-cause mortality, hyperlipidemia population, hypertensive population

## Abstract

**Context:**

Limited studies have shown a protective effect of very long-chain saturated fatty acids (VLSFAs) on healthy aging, diabetes, heart failure, and risk factors related to cardiovascular disease (CVD), but the role of VLSFAs on mortality risk is unclear.

**Objective:**

We aimed to investigate the association of serum docosanoic acid (C22:0) and serum lignoceric acid (C24:0) with all-cause and disease-specific mortality and to confirm the effect of VLSFAs on mortality risk in the whole, hyperlipidemia, and hypertensive populations.

**Methods:**

A total of 4132 individuals from the 2003-2004, 2011-2012 National Health and Nutrition Examination Survey (NHANES) were included in this study. There were 1326 and 1456 participants in the hyperlipidemia and hypertensive population, respectively. Mortality information was confirmed using the National Death Index (NDI). Multiple model calibration was performed using Cox regression analysis for known risk factors to explore the association between circulating VLSFAs and all-cause or CVD or coronary heart disease (CHD) mortality.

**Results:**

In the whole population, individuals with higher circulating C22:0 and C24:0 as a percentage of total serum fatty acid levels reduced the risks of mortality of all-cause (C22:0: HR = .409; 95% CI, 0.271-0.618; C24:0: HR = 0.430; 95% CI, 0.283-0.651), CVD (C22:0: HR = 0.286; 95% CI, 0.134-0.612; C24:0: HR = 0.233; 95% CI, 0.101-0.538), and CHD (C22:0: HR = 0.401; 95% CI, 0.187-0.913; C24:0: HR = 0.263; 95% CI, 0.082-0.846). Similar to the whole population, individuals with higher circulating C22:0 and C24:0 as a percentage of total serum fatty acid levels in the hyperlipidemia and hypertensive populations were also protective for all-cause, CHD, and CVD mortality.

**Conclusion:**

Our results confirm the protective effect of high levels of circulating VLSFAs (C22:0 and C24:0) on CVD, CHD, and all causes of death in the whole, hyperlipidemia, and hypertensive populations.

Circulating very long-chain saturated fatty acids (VLSFAs) with a carbon chain length greater than 20 [1], particularly blood cell membrane fatty acids, reflect the quantity and quality of dietary fat intake and influence endogenous lipid metabolism. VLSFAs, as saturated fatty acids, are strongly associated with disease and are also integrated biomarkers of diet and metabolism [2].

Cardiovascular disease (CVD) is the leading cause of death, accounting for 18 million deaths worldwide in 2017. In China, approximately two-fifths of deaths in 2014 were caused by CVD [3]. Dietary saturated fat has been suggested to play an important role in the development of CVD [4]. CVD is a group of heart and vascular diseases [5] that includes coronary heart disease (CHD), which develops when atherosclerotic plaques block the coronary arteries, ischemic heart disease, and stroke. High intakes of saturated fatty acids are associated with elevated cholesterol levels, which are an important precursor of CVD. Results of a meta-analysis have shown [6] that higher concentrations of circulating total saturated fatty acids are associated with an increased risk of CVD, CHD, and stroke. Saturated fatty acids also promote inflammation and oxidative stress [7]; excessive oxidative stress and inflammation contribute to the development of CVD [8]. And replacing dietary carbohydrates with saturated fatty acids increases low-density lipoprotein cholesterol, which is a risk factor for CVD [9]. In contrast, replacing saturated fatty acids with unsaturated fatty acids reduces serum total cholesterol and low-density lipoprotein cholesterol levels and the risk of CVD events [10]. Therefore, saturated fatty acid is generally considered to be detrimental to human health, and limiting saturated fatty acid intake has been considered a key component in reducing the risk of chronic disease.

However, researchers have found that the effects of saturated fatty acids on organismal health tend to become more beneficial as the carbon chain length increases. Previous studies have revealed an inverse association between the proportion of circulating VLSFAs and CVD-related risk factors, such as subclinical inflammation [11], poor metabolic profile [12], and insulin resistance [13]. Higher levels of circulating VLSFAs are associated with a reduced risk of CHD [14], heart failure [2], atrial fibrillation [15], and cardiac arrest [16]. The aforementioned studies suggest a protective effect of VLSFAs on diseases and their risk factors, but do not further investigate the role of VLSFAs in the risk of death from disease. Although the Ludwigshafen Risk and Cardiovascular Health Study [17] analyzed the relationship between VLSFAs and death, no correlation was observed.

Given that current evidence suggests a strong association between VLSFAs and risk of disease development, that the role of VLSFAs on mortality risk is currently unclear, and that no studies of the role of VLSFAs on mortality risk have been conducted in different populations, this study was designed to investigate the effect of serum VLSFAs on CVD, CHD, and all-cause mortality in the whole, hyperlipidemia, and hypertensive populations by analyzing the National Health and Nutrition Examination Survey (NHANES) database from 2003-2004, 2011-2012.

## Materials and Methods

### Study Population

NHANES is a representative multistage, stratified sample health survey in the United States. It consists of an interview and examination component that includes demographic, socioeconomic, dietary, health-related questions, physiological measurements, laboratory tests, and other information administered by trained medical personnel. Detailed information has been provided previously [18, 19]. Briefly, adults (aged ≥18 years) with serum VLSFAs measured in NHANES in 2003-2004, 2011-2012 were selected for our study. After excluding participants with missing values of our VLSFAs and/or mortality information of interest, 4132 participants were included in the study. These 4132 participants represented the whole population, which included the hyperlipidemia (1326 participants) and hypertensive (1456 participants) populations. Institutional review board approval and written informed consent were obtained from the National Center for Health Statistics prior to data collection.

### Ethics Declarations

NHANES is conducted by the Centers for Disease Control and Prevention (CDC) and the National Center for Health Statistics (NCHS). The NHANES study protocol was reviewed and approved by the NCHS Research Ethics Review Committee. All participants in NHANES provided written informed consent.

### Outcome Ascertainment

The outcome is the final mortality status as determined by the National Death Index (NDI) [20]. The NDI is a highly reliable source for identifying mortality. The International Classification of Diseases, tenth revision (ICD-10) was used to identify disease-specific deaths. Deaths due to CVD were defined as ICD-10 codes I00-I09, I11, I13, I20-I51, and I60-I69. Deaths due to CHD were defined as ICD-10 codes I00-I09, I11, I13, and I20-I51. We defined CVD death as death from CVD and CHD death as death from CHD.

### Exposure Measurement

The exposure factors identified in our study were serum docosanoic acid and lignoceric acid. Because fatty acid metabolism depends on the balanced amount of each fatty acid precursor and/or product [21], we expressed the 2 exposure factors as relative values (as percentages of serum total fatty acids) and performed quintiles.

Blood samples were collected and analyzed by the CDC [22]. Esterified fatty acids are hydrolyzed primarily from triglycerides (TGs), phospholipids, and cholesteryl esters by sequential treatment with mineral acid and base in the presence of heat. The extract is derivatized with pentafluorobenzyl bromide (PFBBr) in the presence of triethylamine to form pentafluorobenzyl esters. The reaction mixture is injected onto a capillary gas chromatograph column to resolve individual fatty acids of interest from other matrix constituents. Quantitation is accomplished by comparing the peak area of the analyte in the unknown with the peak area of a known amount in a calibrator solution. Calculations are corrected based on the peak area of the internal standard in the unknown compared with the peak area of the internal standard in the calibrator solution.

### Covariate Assessment

The following covariates were used in our study: age; sex was categorized as male and female; participants’ self-reported race was categorized as Mexican American, non-Hispanic White, non-Hispanic Black, non-Hispanic Asian, and other races; height and weight were assessed by body mass index (BMI), and we calculated participants’ BMI using the following formula: based on weight (kg)/height^2^ (m); smoking status was assessed by the question “Ever smoked at least 100 cigarettes in your lifetime?” and was categorized as yes, no, or don’t know; drinking status was assessed by the question “Have you had at least 12 alcoholic drinks in your lifetime?” and was categorized as yes, no, or don’t know; waist circumference was estimated in centimeters; physical activity was assessed by the question “Have you done any physical activity in the past 7 days?” and was categorized as yes, no, or don’t know; monthly household income was categorized as $0 to $4,999, $5000 to $9,999, $10 000 to $14,999, $15 000 to $19,999, $20 000 to $24 999, and don’t know; the Alternative Healthy Eating Index (AHEI) [23-25] is based on a comprehensive review of the original Healthy Eating Index and subsequent studies that included 11 food components (fruits, vegetables, whole grains, sugary drinks and juices, nuts and legumes, red and processed meats, trans fats, long-chain n-3 fats, polyunsaturated fatty acids, sodium, and alcohol), and showed associations with chronic disease risk and even with total and cause-specific mortality [26]. The scores for each item were calculated by dividing the actual intake by the maximum criterion and multiplying by the full score for that item, with each component of the food ranging from 0 (unhealthy) to 10 (healthiest) and the total score ranging from 0 to 110. Finally, we combined the scores for each of the 11 items to produce the final AHEI score, with higher total scores indicating a healthier diet; systolic and diastolic blood pressure were estimated in mm Hg; fasting blood glucose was estimated in mmol/L; total energy was estimated in kcal; total unsaturated fatty acids was estimated in μmol/L; TGs, total cholesterol, and high-density lipoprotein (HDL) cholesterol were estimated in mmol/L.

### Statistical Analysis

The associations between serum C22:0 and C24:0 and CVD, CHD, and all-cause mortality were examined in the whole, hyperlipidemia, and hypertensive populations by using Cox proportional-risk regression models with the following covariates: age, sex, and race/ethnicity (model 1); model *z* smoking status, alcohol consumption status, waist circumference, physical activity, household income, and AHEI (model 2); model 2 + systolic blood pressure, diastolic blood pressure, fasting glucose, total energy, total unsaturated fatty acids, TGs, total cholesterol, and HDL cholesterol (model 3); and model 3 + serum stearic acid (C16:0)/palmitic acid (C18:0) (model 4). The covariates corrected by the restricted cubic splines and sensitivity analysis were model 3. The covariates corrected by the Cox proportional-risk regression analysis between serum C18:0 and CVD, CHD, and all-cause mortality were model 3 + serum C22:0 and C24:0 (model 5). The results of the trend test were expressed as *P* trend. Model assumptions were checked for all analyses. All statistical analyses were performed using the survey module of SPSS software version 25.0. A 2-tailed *P* value of less than .05 was considered statistically significant.

## Results

### Characteristics of Participants

There were 4132 participants (2095 women), 1326 participants (654 women), and 1456 participants (736 women) in the whole, hyperlipidemia, and hypertensive populations. The mean (SD) age of the participants was 48.6 years (18.9), 58.6 years (15.2), and 59.7 years (15.8) in the whole, hyperlipidemia, and hypertensive populations.

In the whole population, individuals with higher serum C22:0 (Table 1) were more likely to be younger; female; non-Hispanic White and Black; have higher BMI; nonsmoker; nondrinker; smaller waist circumference; be physically inactive; have moderate to high income; have higher AHEI scores; have lower systolic blood pressure, fasting glucose, and TGs; and have higher total unsaturated fatty acids and HDL cholesterol. Individuals with higher serum C24:0 (Table 2) were more likely to be younger; non-Hispanic White and Black; have lower BMI; have smaller waist circumference; be physically inactive; have moderate to high income; have higher AHEI scores; have lower systolic blood pressure, fasting glucose, and TGs; and have higher total energy, unsaturated fatty acids, cholesterol, and HDL cholesterol. In addition, the characteristics of participants in the hyperlipidemia (Supplementary Tables S1 and S2) [27] and hypertensive (Supplementary Tables S3 and S4) [27] populations were basically similar to those of the whole population, with slight differences in BMI, smoking, drinking, and blood pressure.

**Table 1.** Baseline characteristics of the whole population based on the percentage of docosanoic acid in serum fatty acids

Variables	Total	Quartile 1	Quartile 2	Quartile 3	Quartile 4	Quartile 5	*P*
	N = 4132	n = 809	n = 808	n = 809	n = 808	n = 809	
Age, y	48.6 ± 18.9	50.6 ± 18.9	50.7 ± 19.4	47.3 ± 18.9	47.2 ± 18.8	46.6 ± 17.8	<.001
Sex, female, n,%	2095 (50.7)	326 (40.3)	395 (48.9)	407 (50.3)	433 (53.6)	489 (60.4)	<.001
Race, n (%)							<.001
Mexican American	607 (14.7)	169 (20.9)	140 (17.3)	134 (16.6)	90 (11.1)	61 (7.5)	
Non-Hispanic White	1905 (46.1)	318 (39.3)	370 (45.8)	367 (45.4)	406 (50.2)	394 (48.7)	
Non-Hispanic Black	874 (21.2)	111 (13.7)	117 (14.5)	150 (18.5)	205 (25.4)	283 (35.0)	
Non-Hispanic Asian	311 (7.5)	89 (11.0)	77 (9.5)	68( 8.4)	49 (6.1)	27 (3.3)	
Other races	435 (10.5)	122 (15.1)	104 (12.9)	90 (11.1)	58 (7.2)	44 (5.5)	
Body mass index	28.6 ± 6.6	29.1 ± 6.6	28.4 ± 6.3	28.1 ± 6.4	28.4 ± 6.7	29.3 ± 6.9	<.001
Smoking status, n (%)							.007
Yes	955 (23.1)	230 (28.4)	177 (21.9)	185 (22.9)	187 (23.1)	158 (19.5)	
No	2926 (70.8)	528 (65.3)	580 (71.8)	574 (71.0)	579 (71.7)	602 (74.4)	
Don’t known	251 (6.1)	51 (6.3)	51 (6.3)	50 (6.2)	42 (5.2)	49 (6.1)	
Drinking status, n (%)							.025
Yes	2787 (67.4)	569(70.3)	523 (64.7)	558 (69.0)	566 (70.0)	514 (63.5)	
No	1096 (26.5)	189(23.4)	236 (29.2)	201 (24.8)	200 (24.8)	246 (30.4)	
Don’t known	249 (6.0)	51(6.3)	49 (6.1)	50 (6.2)	42 (5.2)	49 (6.1)	
Waist circumference, cm	98.2 ± 15.8	101.1 ± 16.5	98.3 ± 15.3	96.5 ± 15.6	96.8 ± 15.3	98.3 ± 15.9	<.001
Physical activity, n (%)							<.001
Yes	2133 (51.6)	451 (55.7)	427 (52.8)	431 (53.3)	416 (51.5)	368 (45.5)	
No	1532 (37.1)	253 (31.3)	268 (33.2)	284 (35.1)	309 (38.2)	374 (46.2)	
Don’t known	467 (11.3)	105 (13.0)	113 (14.0)	94 (11.6)	83 (10.3)	67 (8.3)	
Income status, n (%), $							.002
0-4999	949 (23.0)	218 (26.9)	193 (23.9)	188 (23.2)	168 (20.8)	161 (19.9)	
5000-9999	1323 (32.0)	270 (33.4)	253 (31.3)	271 (33.5)	256 (31.7)	242 (29.9)	
10 000-14 999	722 (17.5)	139 (17.2)	136 (16.8)	125 (15.5)	144 (17.8)	162 (20.0)	
15 000-19 999	541 (13.1)	74 (9.1)	102 (12.6)	99 (12.2)	117 (14.5)	136 (16.8)	
20 000-24 999	374 (9.1)	66 (8.2)	75 (9.3)	78 (9.6)	84 (10.4)	67 (8.3)	
Don’t known	223 (5.4)	42 (5.2)	49 (6.1)	48 (5.9)	39 (4.8)	41 (5.1)	
AHEI	53.3 ± 13.7	52.0 ± 14.2	53.2 ± 14.4	53.8 ± 13.4	53.3 ± 13.7	54.0 ± 12.9	.036
Systolic blood pressure, mm Hg	123.5 ± 19.6	126.6 ± 21.0	125.2 ± 19.8	123.2 ± 20.0	121.6 ± 18.5	121.7 ± 18.5	<.001
Diastolic blood pressure, mm Hg	69.5 ± 13.1	69.8 ± 14.0	69.4 ± 13.2	69.3 ± 13.3	68.9 ± 12.5	70.6 ± 11.9	.055
Fasting glucose, mmol/L	5.9 ± 1.8	6.4 ± 2.4	5.9 ± 1.9	5.7 ± 1.5	5.7 ± 1.6	5.5 ± 1.3	<.001
Total energy, kcal	2083.9 ± 858.9	2054.9 ± 856.7	2070.9 ± 840.1	2109.6 ± 864.7	2081.5 ± 889.1	2096.3 ± 844.8	.737
Total unsaturated fatty acids, μmol/L	28.4 ± 14.9	26.4 ± 14.3	27.4 ± 14.3	28.5 ± 14.7	29.0 ± 15.5	30.9 ± 15.6	<.001
Triglycerides, mmol/L	1.5 ± 1.2	2.5 ± 1.8	1.6 ± 0.8	1.3 ± 0.6	1.2 ± 0.5	1.0 ± 0.5	<0.001
Total cholesterol, mmol/L	5.1 ± 1.1	5.0 ± 1.2	5.0 ± 1.2	5.0 ± 1.0	5.0 ± 1.0	5.1 ± 1.0	.051
High-density lipoprotein cholesterol, mmol/L	1.4 ± 0.4	1.2 ± 0.4	1.4 ± 0.4	1.4 ± 0.4	1.4 ± 0.4	1.5 ± 0.4	<.001

Continuous variables in the baseline data were analyzed using analysis of variance, expressed as mean ± SD, and categorical variables were expressed as number of samples (frequency) using chi-square tests.

Abbreviation: AHEI, Alternative Healthy Eating Index.

**Table 2.** Baseline characteristics of the whole population based on the percentage of lignoceric acid in serum fatty acids

Variables	Total	Quartile 1	Quartile 2	Quartile 3	Quartile 4	Quartile 5	*P*
	N = 4132	n = 811	n = 810	n = 811	n = 810	n = 810	
Age, y	48.6 ± 18.9	52.4 ± 19.4	49.7 ± 18.8	48.2 ± 19.1	46.0 ± 18.5	46.3 ± 17.8	<.001
Sex, female, n, %	2095 (50.7)	400 (49.3)	434 (53.6)	414 (51.0)	394 (48.6)	410 (50.6)	.318
Race, n (%)							<.001
Mexican American	607 (14.7)	187 (23.1)	136 (16.8)	104 (12.8)	86 (10.6)	71 (8.8)	
Non-Hispanic White	1905 (46.1)	353 (43.5)	363 (44.8)	412 (50.8)	371 (45.8)	375 (46.3)	
Non-Hispanic Black	874 (21.2)	102 (12.6)	128 (15.8)	145 (17.9)	213 (26.3)	274(33.8)	
Non-Hispanic Asian	311 (7.5)	65 (8.0)	69 (8.5)	67 (8.3)	67 (8.3)	42 (5.2)	
Other races	435 (10.5)	104 (23.7)	114 (14.0)	83 (10.3)	73 (9.0)	48 (6.0)	
Body mass index	28.6 ± 6.6	30.4 ± 6.7	28.9 ± 6.7	27.9 ± 6.3	27.8 ± 6.4	28.1 ± 6.3	<.001
Smoking status, n (%)							.523
Yes	955 (23.1)	179 (22.1)	193 (23.8)	196 (24.2)	197 (24.3)	171 (21.1)	
No	2926 (70.8)	583(71.9)	566 (69.9)	568 (70.0)	556 (68.6)	597 (73.7)	
Don’t known	251 (6.1)	4 9(6.0)	51 (6.3)	47 (5.8)	57 (7.0)	42 (5.2)	
Drinking status, n (%)							.121
Yes	2787 (67.4)	522 (64.4)	534 (65.9)	553 (68.2)	568 (70.1)	556 (68.6)	
No	1096 (26.5)	240 (29.6)	227 (28.0)	211 (26.0)	185 (22.8)	212 (26.2)	
Don’t known	249 (6.0)	49 (6.0)	49 (6.0)	47 (5.8)	57 (7.0)	42 (5.2)	
Waist circumference, cm	98.2 ± 15.8	104.2 ± 16.2	98.7 ± 15.3	96.8 ± 15.2	95.4 ± 15.2	96.0 ± 15.3	<.001
Physical activity, n (%)							.012
Yes	2133 (51.6)	430 (53.0)	417 (51.5)	422 (52.0)	435 (53.7)	393 (48.5)	
No	1532 (37.1)	277 (34.2)	288 (35.6)	295 (36.4)	290 (35.8)	344 (42.5)	
Don’t known	467 (11.3)	104 (12.8)	105 (13.0)	94 (11.6)	85 (10.5)	73 (9.0)	
Income status, n (%), $							<.001
0-4999	94 9(23.0)	223 (27.5)	202 (24.9)	196 (24.2)	167 (20.6)	146 (18.0)	
5000-9999	1323 (32.0)	280 (34.5)	257 (31.7)	261 (32.2)	256 (31.6)	244 (30.1)	
10 000-14 999	722 (17.5)	140 (17.3)	135 (16.7)	135 (16.6)	144 (17.8)	155 (19.1)	
15 000-19 999	541 (13.1)	81 (10.0)	106 (13.1)	103 (12.7)	103 (12.7)	135 (16.7)	
20 000-24 999	374 (9.1)	51 (6.3)	59 (7.3)	74 (9.1)	97 (12.0)	89 (11.0)	
Don’t known	223 (5.4)	36 (4.4)	51 (6.3)	42 (5.2)	43 (5.3)	41 (5.1)	
AHEI	53.3 ± 13.7	51.4 ± 14.3	53.3 ± 13.7	53.3 ± 13.6	53.4 ± 13.6	55.0 ± 13.2	<.001
Systolic blood pressure, mm Hg	123.5 ± 19.6	126.4 ± 21.2	125.0 ± 20.2	123.6 ± 19.9	121.2 ± 17.9	121.8 ± 18.2	<.001
Diastolic blood pressure, mm Hg	69.5 ± 13.1	69.3 ± 14.0	70.0 ± 12.8	68.9 ± 13.6	69.1 ± 12.5	70.5 ± 12.3	.085
Fasting glucose, mmol/L	5.9 ± 1.8	6.5 ± 2.6	5.8 ± 1.6	5.7 ± 1.4	5.6 ± 1.4	5.6 ± 1.5	<.001
Total energy, kcal	2083.9 ± 858.9	1970.4 ± 798.6	2063.7 ± 833.3	2093.5 ± 880.1	2100.1 ± 898.4	2188.4 ± 881.5	<.001
Total unsaturated fatty acids, μmol/L	28.4 ± 14.9	26.0 ± 14.0	27.6 ± 13.8	28.3 ± 14.9	28.5 ± 15.0	31.7 ± 16.4	<.001
Triglycerides, mmol/L	1.5 ± 1.2	2.7 ± 1.8	1.6 ± 0.8	1.3 ± 0.6	1.1 ± 0.5	0.9 ± 0.4	<.001
Total cholesterol, mmol/L	5.1 ± 1.1	5.0 ± 1.2	5.0 ± 1.2	5.1 ± 1.0	5.0 ± 1.0	5.2 ± 1.0	.012
High-density lipoprotein cholesterol, mmol/L	1.4 ± .4	1.2 ± 0.4	1.4 ± 0.4	1.4 ± 0.4	1.5 ± 0.4	1.5 ± 0.4	<.001

Continuous variables in the baseline data were analyzed using analysis of variance, expressed as mean ± SD, and categorical variables were expressed as number of samples (frequency) using chi-square tests.

Abbreviation: AHEI, Alternative Healthy Eating Index.

### Mortality Risk in Relation To Serum Docosanoic Acid and Serum Lignoceric Acid

In the whole population, individuals with higher serum C22:0 and C24:0 levels (see Tables 3 and 4) had lower risks of dying of CVD and CHD. After adjustment for age, sex, and race, individuals with higher serum C22:0 had 55.8%, 68.9%, and 71.5% lower risks of all-cause, CHD, and CVD mortality, respectively. After further correction for lifestyle factors, the risks of all-cause, CHD, and CVD mortality were reduced by 52.8%, 59.9% (*P* = .027), and 69.9%, respectively. In model 3, there were 59.1% (*P* < .001) and 71.4% (*P* = .001) reductions in the risks of all-cause and CVD mortality, respectively. Individuals with higher serum C24:0 had 53.0%, 77.9%, and 75.0% lower risks of all-cause, CHD, and CVD mortality, respectively, after adjusting for age, sex, and race. In model 2, the risks of all-cause, CHD, and CVD mortality were reduced by 46.9%, 68.3%, and 71.3%, respectively. In model 3 all-cause, CHD and CVD mortality risks were reduced by 57.0% (*P* < .001), 73.7% (*P* = .025), and 76.7% (*P* = .001), respectively.

**Table 3.** Association of the percentage of docosanoic acid in serum fatty acids with all-cause, coronary heart disease, and cardiovascular disease mortality in the whole population

	Quartile 1	Quartile 2		Quartile 3		Quartile 4		Quartile 5		*P* trend
	(<0.459%)	(0.459%-0.545%)		(0.545%-0.622%)		(0.622%-0.722%)		(>0.722%)		
		HR (95% CI)	*P*	HR (95% CI)	*P*	HR (95% CI)	*P*	HR (95% CI)	*P*	
All-cause mortality										
Deaths/person-y	122/808	93/807		80/808		74/806		53/807		
Unadjusted	1 (ref)	0.73 8 (0.563-0.966)	.027	0.629 (0.475-0.834)	.001	0.531 (0.397-0.708)	<.001	0.312 (0.226-0.432)	<.001	<.001
Model 1	1 (ref)	0.673 (0.513-0.883)	.004	0.783 (0.590-1.040)	.091	0.663 (0.495-0.887)	.006	0.442 (0.318-0.614)	<.001	<.001
Model 2	1 (ref)	0.716 (0.537-0.955)	.023	0.850 (0.628-1.150)	.292	0.763 (0.564-1.032)	.079	0.472 (0.333-0.670)	<.001	<.001
Model 3	1 (ref)	0.639 (0.467-0.874)	.005	0.718 (0.509-1.012)	.058	0.680 (0.480-0.965)	.031	0.409 (0.271-0.618)	<.001	.001
CHD mortality										
Deaths/person-y	29/809	20/808		14/809		14/808		8/809		
Unadjusted	1 (ref)	0.672 (0.380-1.189)	.172	0.468 (0.247-0.886)	.020	0.431 (0.227-0.815)	.010	0.207 (0.094-0.453)	<.001	<.001
Model 1	1 (ref)	0.619 (0.349-1.097)	.100	0.591 (0.312-1.121)	.108	0.538 (0.283-1.023)	.059	0.311 (0.141-0.689)	.004	.003
Model 2	1 (ref)	0.726 (0.394-1.337)	.304	0.760 (0.386-1.496)	.428	0.657 (0.333-1.298)	.227	0.401 (0.178-0.903)	.027	.032
Model 3	1 (ref)	0.718(0.359-1.436)	.349	0.769 (0.345-1.716)	.521	0.686 (0.308-1.529)	.357	0.453 (0.170-1.208)	.114	.157
CVD mortality										
Deaths/person-y	52/809	34/808		30/809		19/808		14/809		
Unadjusted	1 (ref)	0.634 (0.412-0.977)	.039	0.555 (0.354-0.870)	.010	0.322 (0.190-0.544)	<.001	0.196 (0.109-0.355)	<.001	<.001
Model 1	1 (ref)	0.574 (0.372-0.887)	.012	0.698 (0.444-1.097)	.119	0.406 (0.239-0.689)	.001	0.285 (0.156-0.520)	<.001	<.001
Model 2	1 (ref)	0.631 (0.396-1.004)	.052	0.889 (0.555-1.424)	.625	0.484 (0.278-0.842)	.010	0.301 (0.157-0.577)	<.001	<.001
Model 3	1 (ref)	0.580 (0.346-0.971)	.038	0.785 (0.452-1.364)	.390	0.454 (0.241-0.854)	.014	0.286 (0.134-0.612)	.001	.002

Abbreviations: CHD, coronary heart disease; CVD, cardiovascular disease; HR, hazard ratio; ref, reference.

**Table 4.** Association of the percentage of lignoceric acid in serum fatty acids with all-cause, coronary heart disease, and cardiovascular mortality in the whole population

	Quartile 1	Quartile 2		Quartile 3		Quartile 4		Quartile 5		*P* trend
	(<0.373%)	(0.373%-0.447%)		(0.447%-0.516%)		(0.516%-0.591%)		(>0.591%)		
		HR (95% CI)	*P*	HR (95% CI)	*P*	HR (95% CI)	*P*	HR (95% CI)	*P*	
All-cause mortality										
Deaths/person-y	148/811	92/809		81/808		55/809		49/808		
Unadjusted	1 (ref)	0.624 (0.481-0.810)	<.001	0.575 (0.438-0.754)	<.001	0.391 (0.287-0.533)	<.001	0.323 (0.234-0.446)	<.001	<.001
Model 1	1 (ref)	0.734 (0.564-0.955)	.021	0.740 (0.563-0.973)	.031	0.569 (0.416-0.779)	<.001	0.470 (0.339-0.653)	<.001	<.001
Model 2	1 (ref)	0.751 (0.565-0.999)	.049	0.772 (0.579-1.028)	.077	0.591 (0.425-0.821)	.002	0.531 (0.375-0.753)	<.001	<.001
Model 3	1 (ref)	0.663 (0.485-0.906)	.010	0.697 (0.503-0.965)	.030	0.538 (0.368-0.786)	.001	0.430 (0.283-0.651)	<.001	<.001
CHD mortality										
Deaths/person-y	39/811	16/810		17/811		8/810		6/810		
Unadjusted	1 (ref)	0.413 (0.231-0.739)	.003	0.456 (0.258-0.807)	.007	0.216 (0.101-0.462)	<.001	0.151 (0.064-0.357)	<.001	<.001
Model 1	1 (ref)	0.479 (0.266-0.861)	.014	0.563 (0.317-0.999)	.050	0.309 (0.143-0.665)	.003	0.221 (0.093-0.527)	.001	<.001
Model 2	1 (ref)	0.585 (0.314-1.090)	.091	0.589 (0.318-1.091)	.092	0.318 (0.139-0.729)	.007	0.317 (0.130-0.775)	.012	.001
Model 3	1 (ref)	0.634 (0.315-1.276)	.202	0.593 (0.290-1.213)	.152	0.411 (0.164-1.030)	.058	0.263 (0.082-0.846)	.025	.012
CVD mortality										
Deaths/person-y	70/811	27/810		29/811		14/810		12/810		
Unadjusted	1 (ref)	0.387 (0.248-0.604)	<.001	0.434 (0.282-0.669)	<.001	0.210 (0.118-0.373)	<.001	0.167 (0.091-0.309)	<.001	<.001
Model 1	1 (ref)	0.456 (0.291-0.715)	.001	0.563 (0.363-0.872)	.010	0.311 (0.174-0.555)	<.001	0.250 (0.134-0.464)	<.001	<.001
Model 2	1 (ref)	0.542 (0.337-0.871)	.011	0.621 (0.392-0.984)	.042	0.339( 0.184-0.624)	.001	0.287 (0.144-0.571)	<.001	<.001
Model 3	1 (ref)	0.531 (0.314-0.900)	.019	0.579 (0.340-0.987)	.045	0.381 (0.194-0.750)	.005	0.233 (0.101-0.538)	.001	<.001

Abbreviations: CHD, coronary artery disease; CVD, cardiovascular disease; ref, reference.

**Figure 1.**
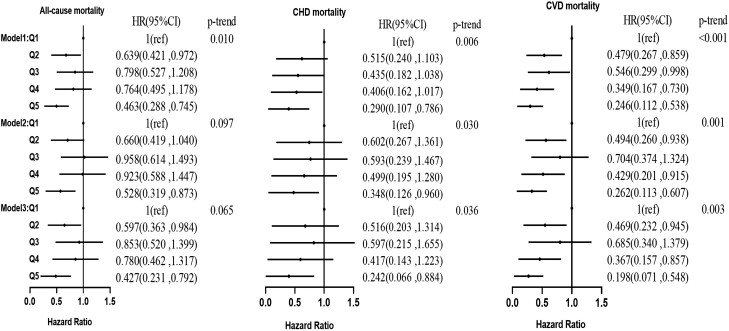
Association of the percentage of docosanoic acid in serum fatty acids with all-cause, CHD, and CVD mortality in the hyperlipidemia population. CHD, coronary heart disease; CVD, cardiovascular disease.

**Figure 2.**
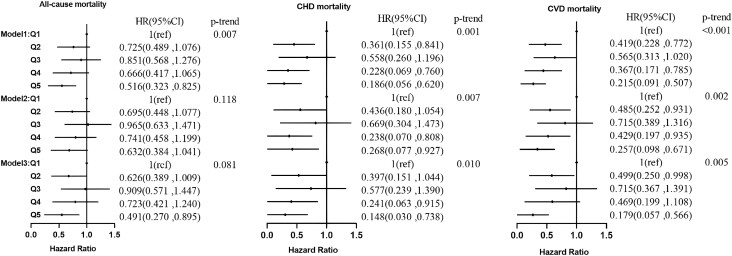
Association of the percentage of lignoceric acid in serum fatty acids with all-cause, CHD, and CVD mortality in the hyperlipidemia population. CHD, is coronary heart disease; CVD, cardiovascular disease.

Similar results were obtained in the hyperlipidemia and hypertensive populations. In the hyperlipidemia population after correcting for age, sex, and race, individuals with higher serum C22:0 (Fig. 1) had 53.7%, 71.0%, and 75.4% lower risks of all-cause, CHD, and CVD mortality, respectively. After further correction for lifestyle, the risks of all-cause, CHD, and CVD mortality were reduced by 47.2%, 65.2%, and 73.8%, respectively. In model 3, the risks of all-cause, CHD, and CVD mortality were reduced by 57.3% (*P* = .007), 75.8% (*P* = .032), and 80.2% (*P* = .002), respectively. In model 1 (Fig. 2), the risks of all-cause, CHD, and CVD mortality were reduced in individuals with higher serum C24:0 by 48.4%, 81.4%, and 78.5%, respectively. In model 2, the risks of CHD and CVD mortality were reduced by 73.2% and 74.3%, respectively. In model 3, the risks of all-cause, CHD, and CVD mortality were reduced by 50.9% (*P* = .020), 85.2% (*P* = .020), and 82.1% (*P* = .003), respectively.

**Figure 3.**
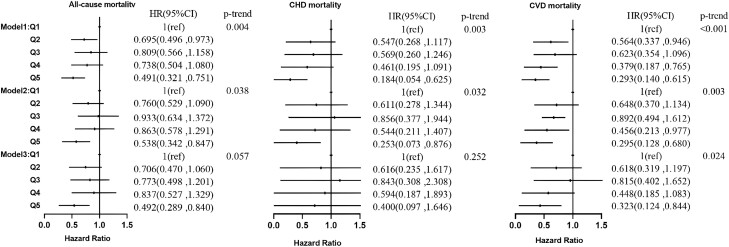
Association of the percentage of docosanoic acid in serum fatty acids with all-cause, CHD, and CVD mortality in the hypertensive population. CHD, coronary heart disease; CVD, cardiovascular disease.

**Figure 4.**
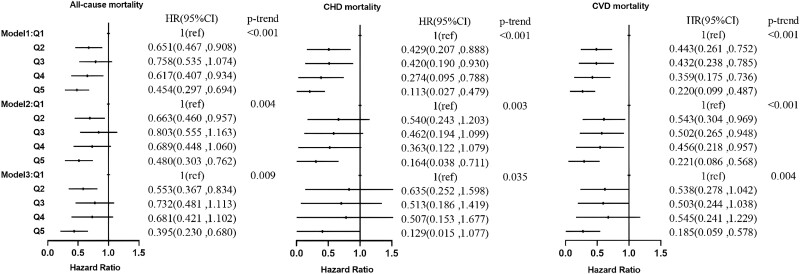
Association of the percentage of lignoceric acid in serum fatty acids with all-cause, CHD, and CVD mortality in the hypertensive population. CHD, coronary heart disease; CVD, cardiovascular disease.

In the hypertensive population, individuals with higher serum C22:0 (Fig. 3) adjusted for age, sex, and race had 50.9%, 81.6%, and 70.7% lower risks of all-cause, CHD, and CVD mortality, respectively. In model 2, the risks of all-cause, CHD, and CVD mortality were reduced by 46.2%, 74.7% (*P* = .030), and 70.5%, respectively. In model 3, the risks of all-cause and CVD mortality were reduced by 50.8% (*P* = .009) and 67.7% (*P* = .021), respectively. Individuals with higher serum C24:0 (Fig. 4) had 54.6%, 88.7%, and 78.0% lower risks of all-cause, CHD, and CVD mortality in model 1. In model 2 the risks of all-cause, CHD, and CVD mortality were reduced by 52.0%, 83.6% (*P* = .016), and 77.9%, respectively. In model 3 all-cause and CVD mortality risks were reduced by 60.5% (*P* = .001) and 81.5% (*P* = .004), respectively.

### Nonlinear Trend Testing

To flexibly model the association of serum C22:0 and C24:0 with CHD, CVD and all-cause mortality, we produced restricted cubic splines. The results showed that higher levels of C22:0 and C24:0 were protective against CHD, CVD, and all-cause mortality, which was consistent with the results of the Cox regression analysis. In the whole population (Supplementary Figs. S1 and S2) [27], higher serum C22:0 was approximately linearly associated with lower risks of all-cause (*P*-linear = .0001; *P*-nonlinear = .8941), CHD (*P*-linear = .2773; *P*-nonlinear = .1703), and CVD (*P*-linear = .0098; *P*-nonlinear = .5427) mortality. The risks of all-cause (*P*-linear = .0001; *P*-nonlinear = .8638), CHD (*P*-linear = .2529; *P*-nonlinear = .4333) and CVD (*P*-linear = .0067; *P*-nonlinear = .9473) mortality tended to decrease with increasing serum C24:0. Similarly, in the hyperlipidemia (Supplementary Figs. S3 and S4) [27] and hypertensive (Supplementary Figs. S5 and S6) [27] populations, higher serum C22:0 and C24:0 were also approximately linearly associated with lower risks of all-cause, CHD, and CVD mortality.

### Sensitivity Analysis

To assess the stability and reliability of the primary results, we analyzed the association of C22:0 and C24:0 with mortality risk by sex.

In the whole population (Supplementary Table S5) [27], in the male subgroup, individuals with higher serum C22:0 had 56.7% and 72.2% reduced risks of all-cause (*P* = .002) and CVD mortality (*P* = .011), respectively. Individuals with higher serum C24:0 had 56.6%, 85.4%, and 79.3% reduced risks of all-cause (*P* = .002), CHD (*P* = .017), and CVD mortality (*P* = .003), respectively. In the female subgroup, individuals with higher serum C22:0 had 63.1% and 75.0% reduction in the risks of all-cause (*P* = .003) and CVD mortality (*P* = .038), respectively. Individuals with higher serum C24:0 had 61.1% and 76.3% reduction in the risks of all-cause (*P* = .007) and CVD mortality (*P* = .045), respectively.

In the hyperlipidemia population (Supplementary Table S6) [27], in the male subgroup, individuals with higher serum C22:0 had 81.5% and 70.4% reduced risks of CHD (*P* = .031) and CVD mortality (*P* = .045), respectively. Individuals with higher serum C24:0 had 65.6%, 83.5%, and 73.0% reduced risks of all-cause (*P* = .008), CHD (*P* = .032), and CVD mortality (*P* = .031), respectively. In the female subgroup, individuals with higher serum C22:0 had 82.3% and 96.6% reduction in the risks of all-cause (*P* = .002) and CVD mortality (*P* = .008), respectively. Individuals with higher serum C24:0 had a 66.9% reduction in the risk of all-cause mortality (*P* = .048), respectively.

In the hypertensive population (Supplementary Table S7) [27], in the male subgroup, individuals with higher serum C24:0 had 61.7% and 83.5% reduced risks of all-cause (*P* = .009) and CVD mortality (*P* = .028), respectively. In the female subgroup, individuals with higher serum C24:0 had 66.6% and 84.0% reduction in the risks of all-cause (*P* = .014) and CVD mortality (*P* = .033), respectively. The protective effect of the serum C22:0 against mortality was attenuated after stratification.

### Correlation Analysis

Previous studies have shown that C16:0 can be extended to C18:0. C18:0 can be endogenously synthesized into VLSFAs. VLSFAs can affect serum TGs and HDL. Surprisingly, our analysis results are consistent with previous studies. In the whole (Supplementary Fig. S7) [27], hyperlipidemia (Supplementary Fig. S8) [27], and hypertensive (Supplementary Fig. S9) [27] populations, serum C18:0 increased with increasing serum C16:0. Total serum VLSFAs were also positively correlated with serum C18:0. As shown in Supplementary Table S8 [27], in the whole, hyperlipidemia, and hypertensive populations, there was a decreasing trend in TGs and increasing trend in HDL with increasing serum C22:0 and C24:0.

### Mortality Risk in Relation to Serum Stearic Acid or Serum Palmitic Acid

Previous studies have shown that C16:0 and C18:0 have adverse effects on the body. Instead, as shown in Supplementary Tables S9 and S10 [27], individuals with higher serum C16:0 or C18:0 in the whole population had no effect on the risks of all-cause, CHD, and CVD mortality. After further correction for C16:0 (Supplementary Table S11) [27], the results were consistent with the Cox regression analysis in model 3. In the whole population, individuals with higher serum C22:0 had 60.4%, 63.7%, and 74.2% lower risks of all-cause, CHD, and CVD mortality, respectively. Individuals with higher serum C24:0 had 57.8%, 78.0%, and 78.5% lower risks of all-cause, CHD, and CVD mortality, respectively. Similarly, after further correction for C18:0 (Supplementary Table S12) [27], in the whole population, individuals with higher serum C22:0 had 59.6% and 71.9% lower risks of all-cause and CVD mortality, respectively. Individuals with higher serum C24:0 had 57.6%, 74.7%, and 77.2% lower risks of all-cause, CHD, and CVD mortality, respectively. The same results were obtained in the hyperlipidemia and hypertensive populations.

## Discussion

In this prospective cohort study from NHANES, our results demonstrate the protective effect of high levels of circulating VLSFAs on all-cause, CHD, and CVD mortality. Furthermore, the risk of all 3 mortality outcomes decreased with increasing levels of VLSFAs. Our results were consistent in the whole, hyperlipidemia, and hypertensive populations.

VLSFAs are saturated fatty acids with a carbon chain length of 20 or more and are derived from diet and endogenous metabolism. Small amounts of VLSFAs have been found in some nuts and seeds and their oils [28], with peanut, macadamia, and rapeseed oils having the highest total VLSFAs. Peanuts and macadamia nut intake is positively correlated with C22:0 and C24:0. A National Institutes of Health-American Association of Retired Persons (NHI-AAPP) Nutrition and Health Study (n = 566 398) showed [29] a significant negative association between nut intake and all-cause mortality (hazard ratio [HR]: 0.78; 95% CI, 0.76-0.81). A prospective cohort study [30] based on a Japanese population (n = 31 552) showed that total nut and peanut intake was negatively associated with all-cause mortality in men (HR: 0.85; 95% CI, 0.75-0.96) and cardiovascular disease mortality in women (HR: 0.72; 95% CI, 0.56-0.92). In addition, dietary intervention studies have shown that circulating levels of VLSFAs can be increased by short-term feeding trials with peanuts and macadamia nuts [31]. Therefore, we suggest that these foods may be appropriately adapted or added to diets of hyperlipidemia, hypertensive, and/or whole populations to prevent or reduce the risk of CVD, CHD, and/or all-cause mortality. The protective effect of VLSFAs on mortality risk can also be taken into account when designing special medical foods for specific populations such as hyperlipidemia and hypertensive individuals, and the proportion of VLSFA-related foods can be adjusted accordingly.

In vivo [32], VLSFAs can be endogenously synthesized from C18:0. C18:0 itself can be derived from C16:0 (Supplementary Figs. S7-S9) [27]. The LSFAs C18:0 and C16:0 have adverse effects on the body. For example, several large cohort studies have shown that C18:0 is associated with a high risk of CVD such as aortic stenosis and venous thrombosis [33]. C16:0 increased the risk of atherosclerosis and CVD [4] and was associated with a higher risk of all-cause mortality [34]. A cohort study reported that replacing C18:0 and C16:0 with plant proteins reduced the risk of myocardial infarction [35]. Surprisingly, our study found no effect of C16:0 or C18:0 on CHD, CVD, and all-cause mortality in the 3 populations (Supplementary Tables S9 and S10) [27]. Furthermore, after correction for C16:0 or C18:0, C22:0 and C24:0 still had stable protective effects on mortality (Supplementary Tables S11 and S12) [27]. We also found that the protective effect of high levels of serum VLSFA on mortality persisted in the hyperlipidemic, hypertensive, and whole populations after correction for TGs, cholesterol, C16:0, C18:0, and other lipid factors associated with death. These results suggest that serum VLSFAs may act as independent protective factors to reduce the risk of death. However, a true understanding of the mechanism of action requires lipidomic analysis of the full natural molecular structure, as these approaches may point to specific enzymes and/or pathways that explain the observed associations.

A prospective study [36] examined the association between blood VLSFAs and the risk of CVD mortality (C22:0: HR: 0.80; 95% CI: 0.66-0.96; C24:0: HR: 0.72; 95% CI, 0.60-0.87) in 3941 older people. The results were generally consistent with our analysis. An inverse association between total VLSFAs and the risk of incident CHD was also reported in 2 nested case-control studies. One of the studies [14] had 408 participants (C22:0: odds ratio [OR]: 0.44; 95% CI, 0.28-0.69; C24:0: OR: 0.41; 95% CI, 0.25-0.65). The other study [37] had 2027 participants (OR: 0.48; 95% CI, 0.32-0.72). Previous studies have also shown [38] that circulating VLSFAs are beneficially associated with the risk factors related to atherosclerosis and CVD. The protective effect of VLSFAs on mortality risk may be related to the following mechanisms.

On the one hand, there is evidence [39] that VLSFAs are associated with the activation of peroxisome proliferator–activated receptors. Thus, VLSFAs are likely to be associated with increased HDL levels and decreased TG levels. And our correlation analysis (Supplementary Table S8) [27] also confirmed this view. This suggests that VLSFAs are actively involved in the etiology of human metabolic and CVDs by regulating peroxisome-related functions and interacting with peroxisome proliferator–activated receptors, thereby reducing the risk of disease morbidity and mortality.

On the other hand, the potential mechanism may be related to the effect of VLSFAs-containing ceramides on apoptosis. VLSFAs are the major components of ceramide and sphingomyelin. The risk of all-cause mortality decreased with increasing plasma ceramide levels at C22:0 and C24:0 and with increasing sphingolipid levels of VLSFAs [40]. The results of several cell culture studies and animal experiments [38, 41] have shown that ceramides containing VLSFAs play an active role in liver homeostasis, hepatic steatosis, myelin maintenance, and apoptosis by affecting cell membrane properties and cell signaling. Unlike C16:0 ceramides [42], C22:0 and C24:0 ceramides actually prevent apoptosis. Animal experiments have also shown [43] that the hearts of genetically engineered mice with reduced ceramides containing VLSFAs developed fibrosis, increased endoplasmic reticulum stress, and underwent apoptosis. As apoptosis can promote the development of many chronic diseases such as cancer, neurodegenerative diseases, and heart disease [44], it is possible that VLSFAs, by binding to ceramides and sphingolipids, may attenuate apoptosis and thereby better protect against the risk of disease morbidity and mortality.

### Strength and Limitation

The main advantage of this population-based study is that the serum VLSFAs indicator we used is a more objective and accurate indicator of the nutritional status of the organism than the dietary VLSFAs indicator, which relies on recall assessment. In addition, we were able to control for potential confounding by various demographic, socioeconomic, lifestyle, and dietary factors using the comprehensive data collected in NHANES 2003-2004, 2011-2012. Although the associations reported in our study were also relatively robust after adjustment for a number of established significant confounders, we cannot completely exclude the possibility of residual confounding by unmeasured factors. Indicators of physical activity, smoking status, and drinking status are categorized only as “yes,” “no,” or “don’t known,” with no specific intensity given. We examined the protective effect of VLSFAs on mortality risk in 3 different populations, in which hyperlipidemia and hypertension were strongly associated with CVD mortality with stable results, but the specific mechanism of the protective effect of VLSFAs on mortality needs further investigation.

## Conclusion

Our study demonstrates the protective effect of high levels of circulating VLSFAs (C22:0 and C24:0) on all-cause, CHD, and CVD mortality in the whole, hyperlipidemia, and hypertensive populations. Our study supports the benefit of increasing serum VLSFA levels in reducing CVD and CHD mortality.

## Data Availability

The NHANES data set is publicly available at the National Center for Health Statistics of the CDC (https://www.cdc.gov/nchs/nhanes/index.htm.). Supplementary tables and figures for this study are available as shown in “References.”

## Abbreviations

AHEI: Alternative Healthy Eating Index; BMI, body mass index; C16:0, serum stearic acid; C18:0, serum palmitic acid; C22:0, serum docosanoic acid; C24:0, serum lignoceric acid; CDC, Centers for Disease Control and Prevention; CHD, coronary heart disease; CVD, cardiovascular disease; HDL, high-density lipoprotein; HR, hazard ratio; ICD-10, International Classification of Diseases, tenth revision; NCHS, National Center for Health Statistics; NDI, National Death Index; NHANES, National Health and Nutrition Examination Survey; OR, odds ratio; TGs, triglycerides; VLSFA, very long-chain saturated fatty acid
