# Correction to “Association of Circulating Very Long-Chain Saturated Fatty Acids With Cardiovascular Mortality in NHANES 2015 to 2016”

**DOI:** 10.1210/clinem/dgad633

**Published:** 2023-11-02

In the above-named article by Tao X, Liu L, Ma P, Hu J, Ming Z, Dang K, Zhang Y, and Li Y (*J Clin Endo Metab*. 2023; doi: 10.1210/clinem/dgad561, there were typographical errors within the article:

In the article as first published, the title was, “Association of Circulating Very Long-Chain Saturated Fatty Acids With Cardiovascular Mortality in NHANES 2015 to 2016.” It has been corrected to read “Association of Circulating Very Long-Chain Saturated Fatty Acids With Cardiovascular Mortality in NHANES 2003-2004, 2011-2012.”

In the Abstract of the article, under the “Methods” section, the text “A total of 4132 individuals from the 2015 to 2016 National Health and Nutrition Examination Survey (NHANES) were included in this study” has been corrected to “A total of 4132 individuals from the 2003-2004, 2011-2012 National Health and Nutrition Examination Survey (NHANES) were included in this study.”

In the “Introduction” section, the text “Given that current evidence suggests a strong association between VLSFAs and risk of disease development, that the role of VLSFAs on mortality risk is currently unclear, and that no studies of the role of VLSFAs on mortality risk have been conducted in different populations, this study was designed to investigate the effect of serum VLSFAs on CVD, CHD, and all-cause mortality in the whole, hyperlipidemia, and hypertensive populations by analyzing the National Health and Nutrition Examination Survey (NHANES) database from 2015 to 2016” has been corrected to “Given that current evidence suggests a strong association between VLSFAs and risk of disease development, that the role of VLSFAs on mortality risk is currently unclear, and that no studies of the role of VLSFAs on mortality risk have been conducted in different populations, this study was designed to investigate the effect of serum VLSFAs on CVD, CHD, and all-cause mortality in the whole, hyperlipidemia, and hypertensive populations by analyzing the National Health and Nutrition Examination Survey (NHANES) database from 2003-2004, 2011-2012.”

In the “Materials and Methods” section, under “Study Population,” the text “Briefly, adults (aged ≥18 years) with serum VLSFAs measured in NHANES in 2015 to 2016 were selected for our study” has been corrected to “Briefly, adults (aged ≥18 years) with serum VLSFAs measured in NHANES in 2003-2004, 2011-2012 were selected for our study.”

In the “Discussion” section, under “Strength and Limitation,” the text “In addition, we were able to control for potential confounding by various demographic, socioeconomic, lifestyle, and dietary factors using the comprehensive data collected in NHANES 2015 to 2016” was corrected to “In addition, we were able to control for potential confounding by various demographic, socioeconomic, lifestyle, and dietary factors using the comprehensive data collected in NHANES 2003-2004, 2011-2012.”

In the “Acknowledgments” section, the text “This research uses data from the 2015 to 2016 National Health and Nutrition Examination Survey (NHANES 2015-2016). We thank the CDC, the NCHS, the National Health and Nutrition Examination Survey Questionnaire, Hyattsville, Maryland: US Department of Health and Human Services, CDC, for providing these data. And we thank the 2015 to 2016 NHANES participants and staff for their valuable contributions” was corrected to “This research uses data from the 2003-2004, 2011-2012 National Health and Nutrition Examination Survey (NHANES 2003-2004, 2011-2012). We thank the CDC, the NCHS, the National Health and Nutrition Examination Survey Questionnaire, Hyattsville, Maryland: US Department of Health and Human Services, CDC, for providing these data. And we thank the 2003-2004, 2011-2012 NHANES participants and staff for their valuable contributions.”

The article has been corrected online.

doi: 10.1210/clinem/dgad561

